# miRNA Targets: From Prediction Tools to Experimental Validation

**DOI:** 10.3390/mps4010001

**Published:** 2020-12-24

**Authors:** Giulia Riolo, Silvia Cantara, Carlotta Marzocchi, Claudia Ricci

**Affiliations:** Department of Medical, Surgical and Neurological Sciences, University of Siena, 53100 Siena, Italy; riolo@student.unisi.it (G.R.); cantara@unisi.it (S.C.); carlottamarzocchi@libero.it (C.M.)

**Keywords:** miRNA target, prediction tools, machine learning, predictive strategies, experimental validation, validation criteria, high-throughput technologies

## Abstract

MicroRNAs (miRNAs) are post-transcriptional regulators of gene expression in both animals and plants. By pairing to microRNA responsive elements (mREs) on target mRNAs, miRNAs play gene-regulatory roles, producing remarkable changes in several physiological and pathological processes. Thus, the identification of miRNA-mRNA target interactions is fundamental for discovering the regulatory network governed by miRNAs. The best way to achieve this goal is usually by computational prediction followed by experimental validation of these miRNA-mRNA interactions. This review summarizes the key strategies for miRNA target identification. Several tools for computational analysis exist, each with different approaches to predict miRNA targets, and their number is constantly increasing. The major algorithms available for this aim, including Machine Learning methods, are discussed, to provide practical tips for familiarizing with their assumptions and understanding how to interpret the results. Then, all the experimental procedures for verifying the authenticity of the identified miRNA-mRNA target pairs are described, including High-Throughput technologies, in order to find the best approach for miRNA validation. For each strategy, strengths and weaknesses are discussed, to enable users to evaluate and select the right approach for their interests.

## 1. Introduction

Among all RNAs, there are non-coding RNAs (ncRNAs). These are functional RNA molecules that do not code for proteins but can modulate protein levels through post-transcriptional regulation. Non-coding RNA genes consist of highly abundant and functionally important RNAs such as transfer RNA (tRNA) and ribosomal RNA (rRNA), as well as RNAs such as small interfering RNAs (siRNAs) and microRNAs (miRNAs) [1]. Currently, over 35,000 miRNA sequences have been identified in 271 organisms [2].

**Figure 1:**
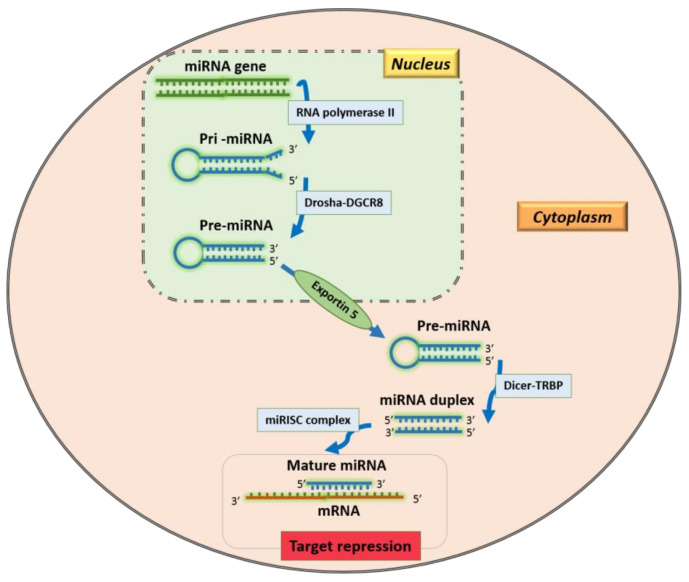
Canonical pathway of miRNA production.

MiRNAs, approximately 18–26 nucleotides long, are expressed in plants, fungi, animals, and unicellular organisms and their synthesis is a finely regulated multi-step process [3]. A primary miRNA (pri-miRNA) is transcribed into the nucleus by an RNA polymerase II. Then, the microprocessor complex, consisting of Drosha and DiGeorge Syndrome Critical Region 8 (DGCR8), cuts a pri-miRNA to generate a precursor-miRNA (pre-miRNA). The pre-miRNA is exported to the cytoplasm by Exportin5/RanGTP and processed by the DICER complex to obtain miRNA-miRNA duplexes [3]. This mechanism is called the “canonical pathway” and represents the main modality of miRNAs production (Figure 1). Other pathways have recently been discovered and can be “Drosha-independent” (i.e., mirtron, t-RNA derived pathways) or “Dicer-independent” (i.e., AGO-, tRNaseZ-dependent pathways) [3].

Whatever the mechanisms, the ultimate goal is the production of a mature, functional miRNA able to regulate protein expression. Bioinformatics analyses have shown that a particular miRNA can regulate expression of up to thousand mRNAs through miRNA-mRNA interaction, and a single mRNA can be controlled by several miRNAs.

Mainly, in mammalian cells, miRNAs bind to the 3′-untranslated region (3′-UTR) of target mRNAs and induce mRNA deadenylation and decapping [4]. Nevertheless, miRNAs can also interact with different regions, such as the 5′-UTR, gene promoter and coding sequence. Pairing with the 3′-UTR was reported to mediate post-transcriptional silencing more effectively than pairing with the 5’-UTR or coding sequence, while miRNA interaction with promoter regions has been shown to induce transcription [4].

However, what are the exact mechanisms of miRNA action? Duplexes of mature miRNA are assembled with an Argonaute (Ago 1–4) protein forming the miRISC complex (miRNA-induced silencing complex). Domain N of Ago fixes miRNA strands in place with the Ago PAZ domain that separates the miRNA duplex. miRISC usually retains the strand with less stable 5’-end base pairing, while the other strand is degraded [4]. The miRNA being partial complementary to a specific mRNA results in translational repression [4]. The process of mRNA silencing also involves other proteins, such as the GW182s [5]. These proteins bridge the link between Ago and downstream effectors, such as the cytoplasmic deadenylase complexes PAN2-PAN3 and CCR4-NOT ensuring mRNA repression. On the other hand, a perfect binding between miRNA-mRNA induces mRNA degradation by enzymes involved in the 5′-to-3′ mRNA decay pathway. In this pathway, poly(A) tail is removed from mRNA, which is then decapped and finally degraded starting from the 5’-end. The GW182 proteins also work as shuttle for Ago2, transferring it from the cytoplasm to the nucleus [5].

Once in the nucleus, miRISC complex can induce nuclear mRNA degradation but also participate to mRNA splicing and chromatin state regulation increasing mRNA levels. It is evident that the biological functions of miRNAs are determined by the mRNAs that they control.

Efficient gene regulation is orchestrated by miRNA localization, target mRNA levels, affinity of the miRNA-mRNA interaction, and the presence of multiple factors (e.g., GW182 proteins). However, miRNA activity on the mRNA targets is difficult to identify.

Despite the growing knowledge about the mechanisms involved in miRNA-mRNA interaction, the identification of potential targets in the human genome is still a challenge, which has been appropriately described as “a classical needle in a haystack problem” [6].

Identification and validation of the interactions taking place between miRNAs and their targets is a critical step for defining the regulatory miRNA role in the complex networks that regulate the biological processes. For any given miRNA, a wide number of potential target sites may be present, and the experimental validation of every potential miRNA target in the laboratory is not feasible, as it is expensive and time-consuming. A computational approach to predict the potential targets of miRNAs can simplify the procedure, allowing an initial selection to reduce the number of target sites to be experimentally validated. Several tools for computational analysis exist, each using a different strategy to predict potential miRNA targets, and their number is constantly increasing. Now, users have the opportunity to access a large range of solutions, but they must decide which tool to use. This choice may not be easy; it is at least necessary to be familiar with the basic assumptions and interpretation of the results.

The aim of this review is to provide practical advice for this challenge, in order to make it easier to understand the subject.

In the first part, we depict the main features employed in miRNA target prediction tools. A specific section is dedicated to the Machine Learning (ML) approach. The strengths and weaknesses of each approach are discussed, to enable the users to evaluate and choose the right tool for their interests. In addition, we include a synthesis of the features of the most common computational tools used for miRNA target prediction. Finally, we provide a stepwise scheme, to facilitate the choice of the best approach to use in practice.

Once the overabundance of information from these algorithms has been collected, specific miRNA-mRNA target interactions are selected to proceed investigating with more efficiency. Four criteria need to be fulfilled in order to confirm a miRNA-mRNA interaction as biologically significant. In the second part of this review, we describe the key approaches and the major experimental procedures to meet the criteria for validation. Most importantly, the advantages and drawbacks of “low-yield” techniques and High-Throughput methods are reported, proving details on why miRNA validation is still challenging.

## 2. miRNA Target Prediction

### 2.1. Predictive Methods

Several approaches underlie the development of miRNA target prediction algorithms. These can be divided into two main categories: algorithms derived from characteristics of the mRNA sequence and/or based on the miRNA-mRNA interaction, and statistical inference based on Machine Learning.

In the first case, different features of the miRNA-target complex are taken into account. Pairings of the seed sequence of miRNAs and mRNAs can be analyzed and evaluated. Thermodynamic analysis can be performed by computing the free energy of pair formation and its thermodynamic stability. The evolutionarily conservation of the target sequence across related species can be assessed. Finally, the 3′-UTR structural accessibility for miRNAs can be evaluated, and the number of miRNA target sites can be calculated, since mRNAs may be regulated by the binding of different miRNAs to multiple target sites.

In the case of Machine Learning, the idea is to identify miRNA targets that reference miRNA-mRNA duplexes with proven biological significance, rather than making “*de novo*” predictions proceeding from sequence features. Machine Learning in general is an application of artificial intelligence that provides systems with the ability to improve automatically through experience; they “learn” from sample datasets and use the acquired information to make predictions on unknown data [7].

The features of these approaches are described in the following sections.

#### 2.1.1. Strategies for *de novo* Predictions

1. Seed pairing

The miRNA sequence is complementary to the sequence of 3′-UTR of potential mRNA targets. The miRNA seed sequence, namely the first 2–7 nucleotides in the miRNA 5′ region, is essential for binding target mRNAs [8]. Indeed, specific characteristics within the seed region, but also within close proximity, have been associated with specific effects on the gene repression induced by miRNAs. Thus, all of these characteristics have been included in target prediction tools [9,10,11].

**Figure 2:**
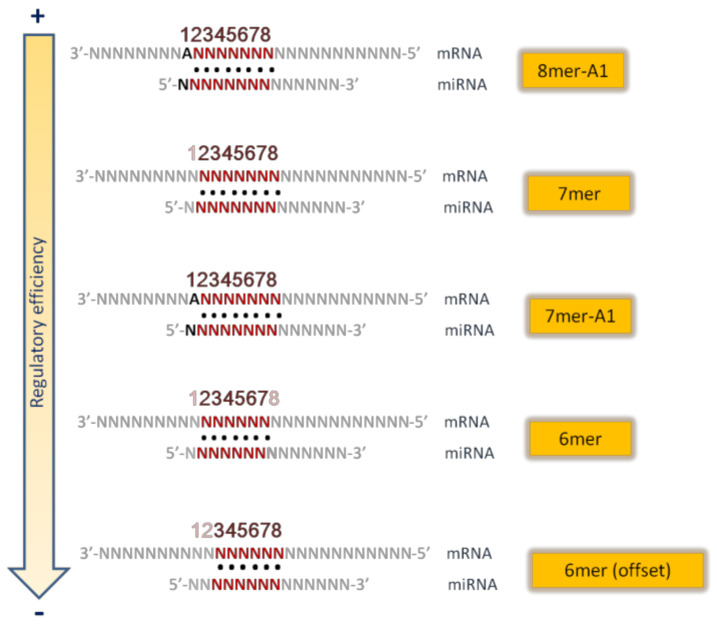
Main types of miRNA seed sequences (in order of descending regulatory efficiency).

The majority of the target prediction algorithms require Watson–Crick pairing between miRNA and mRNA. In other words, guanine (G) must pair with cytosine (C), and adenosine (A) with uracil (U). Different algorithms consider different types of seed matching. The main kinds of seed matches [9,10,12,13] (see Figure 2) are the following:

- 8mer site: a perfect Watson-Crick match from nucleotide 2 to nucleotide 8 of the miRNA seed, with an “A” in mRNA opposite position 1;
- 7mer site: Watson-Crick match from nucleotide 2 to nucleotide 8 without the “A” opposite position 1;
- 7mer A1 site: Watson–Crick match from nucleotide 2 to nucleotide 7 with an A opposite position 1;
- 6mer site: position 2–7 match;
- 6mer site: position 3–8 match.

The presence of an adenosine opposite position 1 of miRNA is important because this adenosine is specifically recognized within the Argonaute protein [11], independently from the nucleotide in the miRNA sequence [14].

2. Thermodynamic stability

Whereas sequence complementarity is clearly informative to predict miRNA–mRNA interactions, important indications for evaluating hybridization stability are provided by the thermodynamic properties of the miRNA-mRNA complexes. In the majority of miRNA target prediction tools, the thermodynamic properties of the miRNA-mRNA complex are assessed by evaluating the free energy of the predicted interaction. Free energy is used to evaluate the stability of a biological system. Though the free energy of a miRNA-mRNA complex is lower, more energy is necessary to break the bond. In other words, when the free energy is low, a miRNA-mRNA complex is estimated to be more thermodynamically stable, and the miRNA-mRNA pairing is considered stronger. This is important information, since a stable miRNA-mRNA interaction can be more likely classified as a real interaction. Nevertheless, it is not easy to establish the proper threshold of free energy, since data obtained from known miRNA-mRNA complexes are insufficient [15].

Some algorithms (e.g., MicroTAR [16]) only assess the thermodynamic stability to validate initial predictions based on sequence complementarities; other algorithms (e.g., RNAhybrid [17]) calculate the free energy as the first step for their evaluation. This strategy can be employed to predict non-canonical binding sites and does not need additional information. However, the algorithm itself is based on free energy calculations obtained from computational models of nucleotide association [18].

To improve prediction efficiency, other features, such as sequence conservation, may be considered to effectively predict miRNA targets.

3. Evolutionary conservation

The analysis of sequence conservation among different species is an approach used to try to reduce the number of false positives obtained by prediction algorithms. The principle underpinning this strategy is that miRNA-mRNA interactions with advantageous biological functions are selected and conserved during evolution. It is worth noting that a higher conservation has been reported in the miRNA seed sequence than in other sequences [9].

Several target prediction algorithms identify orthologous 3′-UTR sequences and then evaluate the conservation of the potential miRNA target site in other species, in particular in those that are closely related (e.g., miRanda [19]). This analysis across the species allows reducing the number of false positive predictions [15]. However, this approach can present some limitations, since it only searches for targets of evolutionary conserved miRNAs, but conservation does not necessarily imply functionality. Indeed, previous studies have reported that about 30% of experimentally validated miRNA targets are not actually conserved [20]. The conservation alone, therefore, is not sufficient for obtaining a reliable miRNA target prediction. Thus, it is strongly recommended to combine this approach with non-conservation models when it is necessary to perform *de novo* predictions.

4. Accessibility of target site

The structural accessibility of the binding sites is another important factor to consider to increase the reliability of predictions. Both miRNA and 3′-UTR of the potential target should be accessible, at least in the regions of the seed sequence. While the accessibility of miRNA seed is ensured, as mature miRNAs are assembled into the RISC [21], the accessibility of the 3′-UTR of mRNA needs to be estimated. Some algorithms identify partially accessible binding sites, then rank the targets in accordance with a specific score [22] or the free energy [23]. Other algorithms calculate the free energy necessary to obtain the accessibility of the complementary site and add it to the energy of hybridization. This sum allows defining which binding sites are accessible and ranking the predicted targets [24].

5. Number of target sites in the same 3′-UTR

It is known that more than one miRNA can bind the same target and, therefore, multiple miRNAs may cooperate in controlling the expression/repression of target genes [25,26]. Therefore, the miRNA-mediated regulation of a specific target gene changes depending on the number of miRNAs binding to that gene [27]. In particular, a relevant element in miRNA-induced silencing seems to be the number and the location of binding sites. Multiple target sites within 10–50 nucleotides of each other usually enhance repression, while sites very close together (< 8 nucleotides) generally operate competitively [14,28]. The use of prediction algorithms also considering multi-targeting significantly reduces the number of predictions, increasing the probability of identifying true targets. Thus, it can be very useful for reducing the number of candidates before performing experimental validation studies of miRNA targets [15].

There are plenty of computational tools that use different strategies or a combination of them.

For example, miRanda, one of the first and most frequently used algorithms, is based on an evaluation of complementarity between miRNAs and 3’-UTR regions, thermodynamic stability of the duplex structure, evolutionary conservation of the entire binding site, and its position within 3′-UTR as a final filter [19].

TargetScan primarily predicts potential miRNA targets by looking for the presence of 8mer, 7mer, and 6mer sites that match the seed region of every miRNA. It is possible to choose to include only conserved sites. Other features, such as seed-pairing stability, 3′ compensatory pairing, and target site abundance, may be evaluated. The putative targets are then ranked based on the predicted efficacy of targeting, calculated using cumulative scores considering all the features of the sites. Moreover, TargetScan predicts the secondary structure to evaluate the free energy of predicted complexes [29].

RNAhybrid finds the minimum free energy for short sequences, but also for the whole miRNA-mRNA complex. This tool evaluates the minimum free energy of hybridization for a long and a short RNA, so the short sequence is hybridized to the best fitting part of the long sequence. The user can impose several restrictions, such as the rate of unpaired bases allowed, to reduce the number of predictions [17].

PITA offers a different approach for miRNA target prediction. The main feature assessed by this program is the accessibility of the target site. It evaluates the free energy gained from miRNA–mRNA pair formation and the energy cost of making the target accessible to the miRNA, and computes the difference between these two parameters. It considers also the “flank sites”, the sites around the seed, which are involved in site accessibility. The user can impose restrictions to reduce the number of resulting targets (i.e., minimum seed size and unpaired bases) [30].

All these tools are available online.

Table 1 summarizes the main features and strategies for these and other largely diffused prediction algorithms.

#### 2.1.2. Machine Learning

The term “Machine Learning” (ML) was used for the first time in 1959 by Arthur Samuel, who described ML as the “field of study that gives computers the ability to learn without being explicitly programmed” [34]. ML methods commonly enable computers to analyze collected data in order to build data-driven models, discover statistically significant patterns and relationships, and consequently make predictions on novel data. In practice, ML algorithms are able to “learn” from sets of data and use the acquired knowledge to analyze similar data and make predictions based on the examples that have been provided [7].

In the case of the miRNA target identification, ML approaches do not refer to miRNA-mRNA features, such as sequence or thermodynamic stability, but try to recognize potential miRNA targets by referring to miRNA–mRNA interactions with proven biological significance. Algorithms are “trained” using experimentally verified miRNA-mRNA interactions as positive examples, and artificially created negative examples. In this way, ML software tries to recognize patterns that discriminate between actual targets and false targets. In the presence of a novel previously unseen dataset, these patterns can be used to correctly predict whether a target is “real” or not.

Interestingly, ML is not programmed to necessitate stringent seed matches in the 3′-UTRs, but it learns by provided examples with biological relevance, so it also allows identifying non-canonical binding sites, including those within the coding regions. On the other hand, ML learns exclusively from the provided examples and consequently it is only able to find results similar to those examples [35].

**Figure 3:**
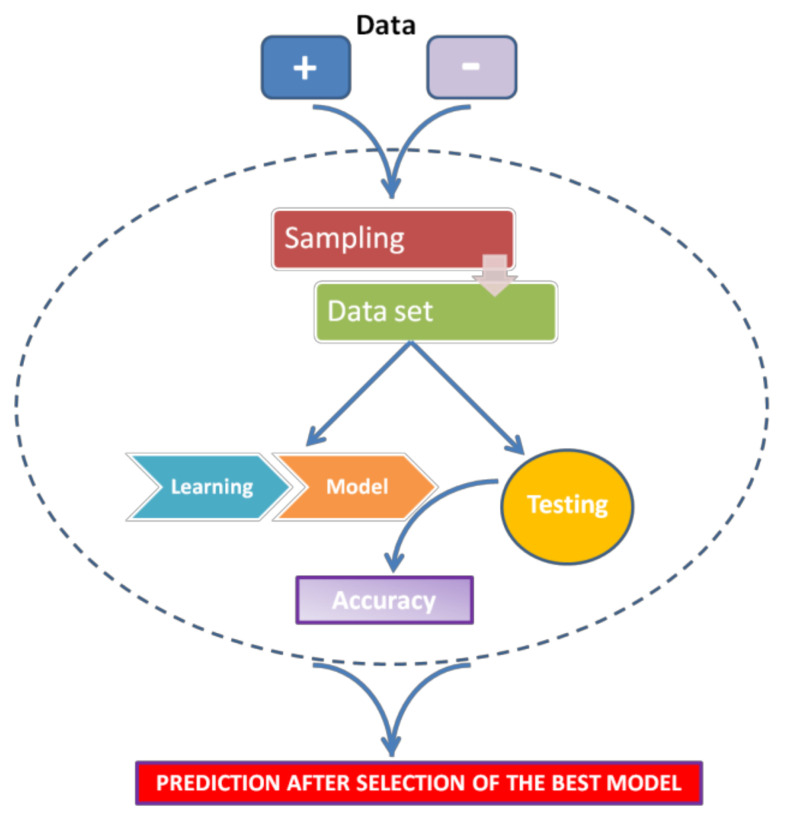
Scheme of Machine Learning general strategy.

The general strategy for ML can be summarized as follows (see Figure 3):

- For every miRNA, identify the presumed binding site from validated mRNA targets (as positive) and non-targets (as negative).
- Extract features from these interactions (regardless of whether they are functional or nonfunctional).
- Train a classifier to discriminate targets from non-targets.
- Test the classifier.
- Use the classifier to sort unknown miRNA-mRNA interactions as positive (target) or negative (non-target).

Some points are of fundamental importance in this process. First, the positive dataset, obtained from experimentally verified databases, largely depends on the features of the database of reference. Several databases of experimentally validated miRNA-mRNA target interactions are available, such as MiRTarBase [36], DIANA-TarBase [37], and miRecords [38]. The use of these validated miRNA-mRNA interactions may introduce some bias, since these data have been produced by different laboratories, using different cell types and approaches [39]. The negative dataset is equally important to obtain a well-trained classifier. If the sequences are too artificial, the Machine-Learning method will probably not be efficiently trained to discriminate between true and false interactions. Conversely, if the negative and the positive datasets are very similar to each other, the ML approach will not be able to distinguish between them. Moreover, an excess of positive or negative dataset can cause underfitting or overfitting models, respectively [35]. Finally, the choice of the validation set is critical for ML: to verify its performance, a model should be assessed on a test dataset that is completely different from the dataset used for the training step (training dataset). A strategy is to exclude a portion of the training set from the training phase and use it as validation set during model optimization [39].

All the ML-based target prediction algorithms are based on a similar set of engineered features and differ mainly in ML architecture and in the experimental datasets that are used.

For example, MBSTar, a prediction tool based on the random forest algorithm, is trained on more than 9000 biologically validated miRNA-mRNA interactions, and approximately 1000 non-interacting pairs, confirmed by data obtained from RISC-associated immunoprecipitation experiments. A combination of 340 sequence and 31 structural features has been applied to an unsupervised learning algorithm to extract 40 putative features for miRNA-mRNA interactions. Different learning classifiers have been analyzed, and the random forest algorithm has displayed the highest accuracy rate when used for target prediction in experimentally validated datasets [40].

Table 2 displays some of the main ML tools used for target prediction, including details about both negative and positive data sets and ML methods. A brief description of the features of such methods is reported in Box 1.

Box 1 **ML Methods: some details.**

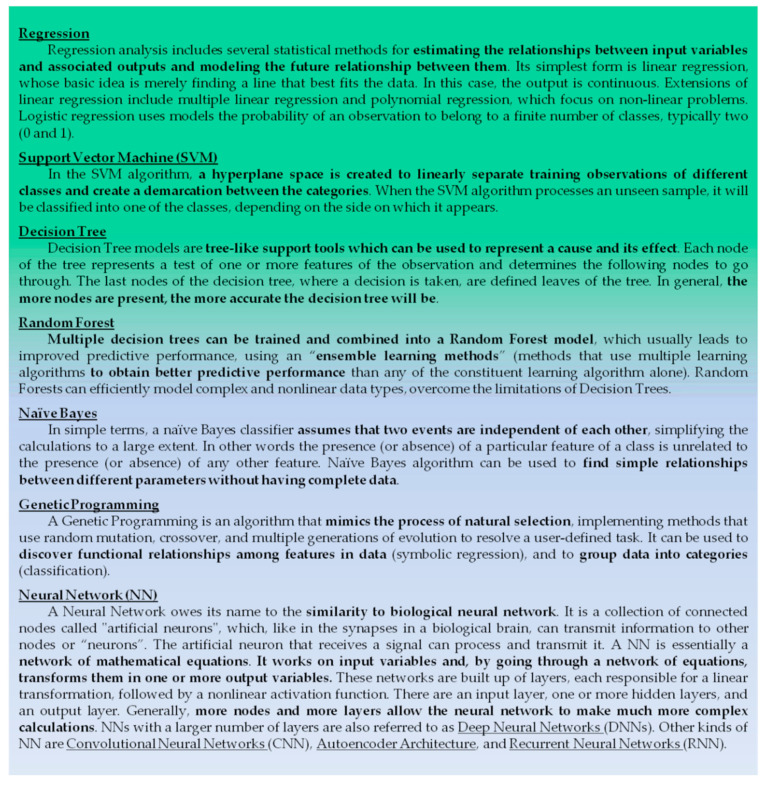

#### 2.1.3. Operative Strategy

The aim of these different approaches is to limit the number of predicted targets and at the same time reduce the loss of true targets. In other words, it is necessary to obtain a compromise between sensitivity and specificity. Some tools may be extremely efficient in identifying true target sites (high sensitivity), but provide a very wide number of predictions (low specificity) [30,48,49]. On the other hand, other tools present high specificity, but quite low sensitivity [10,27,50].

All the available tools have their own predictive strengths and limitations, depending on the strategies they use. A tool exclusively based on seed match, for example, does not consider whether the miRNA-mRNA complex is thermodynamically stable, or the sequence of the target site is evolutionarily conserved and accessible for binding. On the other hand, many non-conserved binding sites in 3′-UTRs have been reported to be functional [51], and such miRNA-mRNA interactions cannot be captured by using only conservation-based prediction tools.

To complicate things, target sites for potential miRNA binding can be deeply affected by other events involving RNA, such as alternative splicing and RNA editing [52,53]. One solution used by several tools to solve this problem is to select either any 3′-UTR or the longest one from available databases (i.e., NCBI’s Refseq or Ensembl). In addition, recent evidence suggests that functional miRNA binding sites are present also within 5′-UTRs and coding sequences, raising the issue of whether it would be useful to extend the analysis to the full-length mRNA [54] and of which binding rules and sequence features should be used in this case [55].

Considering all these observations, the best results to identify miRNA targets are likely to be obtained using a combination of tools that are derived from different prediction assumptions. This approach may allow obtaining a good balance between sensitivity and specificity [56]. Also, in this case, it would be necessary to have the foresight to verify that the prediction tools use the same reference databases and tare regularly updated, since miRNA nomenclature is frequently modified and every year new miRNAs are included in miRBase [15]. One of the many examples of this strategy is the identification of KRAS mRNA as a target for miR-193a-3p in non-small cell lung cancer [57]. In this case, miRNA target prediction was performed using a combination of three different computational algorithms based on different approaches (TargetScan, PicTar, and miRanda), and the results were then confirmed by experimental validation. Thus, the combination of different target prediction algorithms may significantly help researchers in identifying potential miRNA targets, as long as users know the fundamental presumptions, strengths, weaknesses, and limitations of every tool in order to make an informed decision of which ones to use.

**Figure 4:**
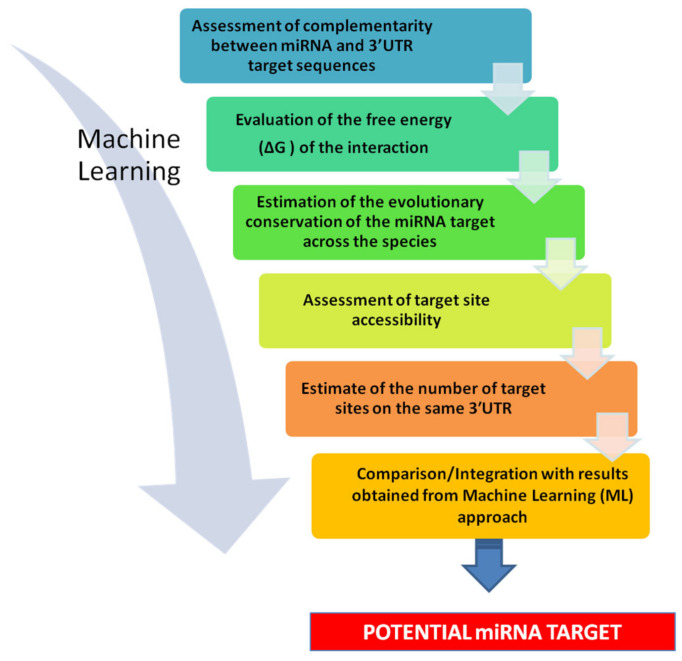
Stepwise strategy for miRNA target prediction. 1) Assessment of complementarity between miRNA and 3′-UTR target sequences. It is advisable to evaluate the score assigned to the prediction, to have an initial indication of its likelihood. 2) Evaluation of the free energy (ΔG) of the interaction. This may give an indication of thermodynamic stability of the miRNA-mRNA interaction and further strengthen predictions. 3) Estimate of the evolutionary conservation of the miRNA target across the species. The more conserved a sequence of DNA, the higher the probability that it corresponds to functional, transcribed, RNA sequences. 4) Assessment of target site accessibility. This may provide a ranking of potential target sites. 5) Estimate of the number of target sites on the same 3′-UTR. The use of multitargeting-based algorithms significantly decreases the number of predicted targets, and raise the chance of identifying true targets. 6) Comparison/Integration with results obtained from Machine Learning (ML) approach. This allows limiting the number of false positive and increasing the strength of the prediction.

A flow-chart summarizing this kind of approach is given in Figure 4.

In addition, data obtained from these computational tools may be coupled and integrated with an ML approach, which can limit the number of false positives and further strengthen the value of the prediction [58]. Since experimental data sets used by ML come from a wide range of experiments, as shown in Table 2, also in this case it is strongly suggested to employ a combination of data sources for ML-based miRNA target prediction in order to reduce the limitations resulting from specific types of data sets. In fact, an ML prediction can only be valid if the data on which it is-based are valid [39]. Such an approach has been successfully used in several cases. An example is the identification of the potential miR-622 targets in breast cancer, using an integrated miRNA prediction process, which included miRanda, miRDB [59], RNA22, TargetScan, and the ML algorithm MiRWalk 3.0 [60]. The intersection of the results obtained from these tools allowed identifying 77 promising targets and constructing a protein-protein interaction network. The results were experimentally validated in vitro, revealing the regulatory mechanisms of miR-622 in breast cancer cells [61].

To avoid getting lost in the sea of available prediction tools, some websites offer interesting solutions to orientate the choice. For example, tools4miRs [62] provides an interactive webpage that allows selecting organisms, prediction approaches, target regions and other features, and gives a list of prediction tools based on the user’s selection.

Another approach was proposed by Kern and colleagues [6], who have set up an interactive webpage, freely available on-line, which helps users perform tool selection by answering a six-step questionnaire based on different criteria.

Box 2 displays some of the main questions to answer for guiding an aware choice of the prediction tools.

Box 2 **Questions to answer for choosing the prediction tool(s).**

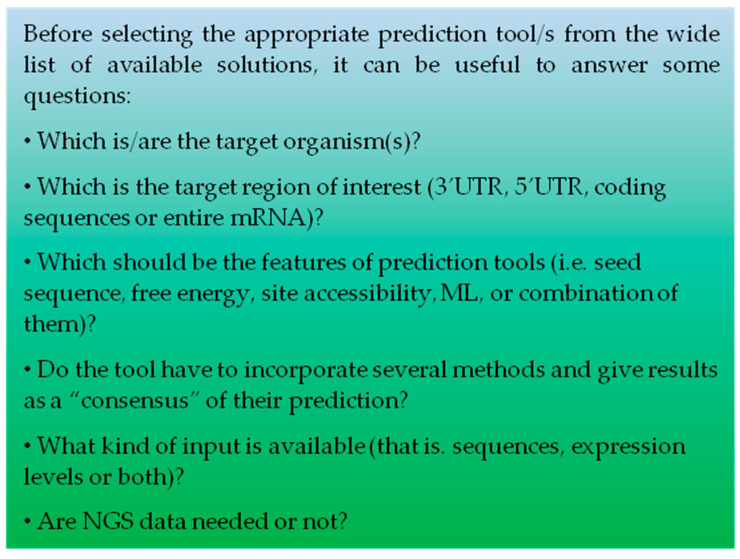

In conclusion, all the tools described may provide important insights about potential miRNA targets. The use of a combinatorial approach may increase the true predictions and limit the number of false positives. However, once bioinformatics analyses have been performed and the potential miRNA targets have been identified, the biological function of predicted miRNA-mRNA interactions needs to be verified and confirmed.

## 3. miRNA Target Validation

### 3.1. Validation Methods

Whichever strategy has been used to predict miRNA-mRNA interactions, candidate miRNA targets should be always verified experimentally to obtain full legitimacy.

Depending on information provided, experimental approaches can be classified into two categories: direct and indirect methods. The first category includes procedures investigating the existence of an interaction between miRNA and its target, by studying directly the miRNA-mRNA pair or introducing a specific target site bound by miRNAs, known as microRNA responsive element (mRE), into a reporter gene, in order to measure potential miRNA-induced changes at protein levels. In particular, these approaches are usually limited to mREs that have been obtained from computational predictions.

On the other hand, the second category includes approaches that observe the effects derived from an altered miRNA expression on mRNA or protein expression, using High-Throughput technologies (i.e., sequencing and mass spectrometry). However, some of these methods suffer from ‘knock-on’ effects. This occurs because, when a miRNA engages its target, it not only induces changes in the protein coded by that gene, but it indirectly produces consequences also on several different genes, whose expression is regulated by the sequence directly targeted by miRNA [63]. As a consequence, the alteration of some products obtained by these approaches is not necessarily to be considered to be resultant from miRNA-mRNA interaction.

As a general approach, the authenticity of the preferred functional miRNA-mRNA target pair, predicted by computational tools, should be validated in the biological model of interest by fulfilling four criteria. First, co-expression of miRNA and predicted target mRNA must be demonstrated. Second, a direct interaction between the miRNA of interest and a specific region within the target mRNA must be proved. Third, gain- and loss-of-function experiments must be performed to demonstrate how miRNAs regulate target protein expression. Fourth, it needs to verify whether the predicted changes in protein expression are associated with modified biological functions [64,65].

#### 3.1.1. Criterion I: Show Co-Expression of miRNA and Target mRNA In Vivo

Co-expression of a miRNA and its target mRNA is the first crucial point required for validation. In a recent study, Cava et al. [66] found that only 19.3% of the total mRNAs is tissue-specific, compared to 39.7% of miRNAs in normal tissues. The co-profiling of miRNAs and mRNAs can allow a direct assessment of whether mRNAs are in part shaped by regulatory miRNAs [67] since co-expressed elements share the same transcriptional program or are regulated by members of the same pathway. Consequently, if they are not co-expressed or co-expression cannot be verified in cells or tissues, there are no reasons to move forward with additional experiments.

Microarray profiling and the latest RNA-sequencing (RNA-seq) represent powerful strategies to carry out large scale studies on the genome. Moreover, Northern blots and quantitative real-time PCR (RT-qPCR), employing nucleic acids extracted from different cell cultures, represent the most exploited methods to demonstrate co-expression [68,69].

Northern blotting is useful, although time-consuming, not quantitative, and suffers from sensitivity issues; RT-qPCR needs low quantities of RNA, taking a short time and low budget to be performed [70]. However, expression analysis by qPCR has several particular challenges, mainly due to the short length and the high similarity of certain miRNAs. A higher number of the available methods analyze mature miRNA levels, although some techniques for detection and quantification of both pri- and pre-miRNAs exist [71]. In addition, appropriate probes (SYBR Green or Taqman) or primers for revealing miRNA and its mRNA target are required. Different approaches employ specific or universal primers and some of them require the use of more primers to increase specificity. Commonly, use of SYBR Green dye represents the most cost-effective solution to quantify mature miRNA levels, although technologies employing probes such as TaqMan are recommended for providing high reproducibility and additional specificity [65,72,73].

When data are too limited to demonstrate the co-expression of miRNA and its target mRNA, it may be necessary to analyze many tissues and/or cell lines. In addition, in situ hybridization and immune-histochemical experiments, using tissues treated with paraffin and fixed in formalin, may be considered useful methods to face the issue of co-expression [64,68,69].

#### 3.1.2. Criterion II: Prove Interaction between miRNA and a Specific mRE Target Site

When co-expression is verified, it is needed to proceed investigating the physical interaction between the miRNA under consideration and the candidate mRE, localized within target mRNA. The current gold standard procedure to prove a direct miRNA-mRNA interaction is the reporter gene assay. In this procedure, 3′-UTR region of the gene of interest, containing the predicted mRE sequence, is cloned immediately downstream of the luciferase gene, or another reporter gene [74], contained in a plasmid. Then, the plasmid must be subcloned under the control of a ubiquitous promoter [75]. At this time, plasmid-containing cells can be co-transfected with miRNA mimics or miRNA inhibitors, in order to perform gain-/loss-of-function experiments. The rationale for performing luciferase assay is based on the evidence that though the mRNA is a real target of miRNA under examination, miRNA mimics, as well as miRNA inhibitors, are able to alter endogenous miRNA concentrations, and probably lead to changes at protein levels [68], as verified by Criterion III.

#### 3.1.3. Criterion III: Demonstrate miRNA-Mediated Effects on Target Protein Expression

After transfection of either miRNA mimics or inhibitors into the cells, protein expression needs to be analyzed. Protein changes can be detected by conventional procedures such as Western blotting, ELISA, and immune-citochemistry experiments [76].

If a given mRNA is a real miRNA target, many changes in levels of proteins should be detected as a consequence of an interaction occurring between them. An increased miRNA activity, deriving from transfection of miRNA mimic into cells expressing the target protein, should decrease target protein expression. On the other hand, a reduced miRNA activity, due to the use of a miRNA inhibitor for cell transfection, should result in increased target protein expression [64,65,68].

Various designs exist for miRNA inhibitors. Locked-nucleic acid (LNA) oligonucleotides are synthetic, modified antisense RNAs. When introduced into cells, these single-stranded molecules perfectly bind to endogenous miRNAs, preventing hybridization with its cellular mRNA targets and so decreasing miRNA activity [15,64]. Similarly, constructs known as ‘sponge’ inhibitors [77] produce RNA sequences containing several sites specific for miRNA in order to sequester endogenous miRNAs and subsequently inhibit their regulatory capacity [78].

In this type of methods, appropriate controls need to be employed to determine the efficiency of transfection. As an example, the transfection of scrambled miRNA sequences should prove the specificity of a miRNA-mRNA interaction [69], and the use of empty vector should demonstrate the efficiency of the transfection [79].

#### 3.1.4. Criterion IV: Demonstrate miRNA Effects on Biological Function

Once criterion III is satisfied, it is finally necessary to demonstrate that protein changes mediated by miRNAs equate to changes in biological function.

On the basis of the target protein, several in vitro and in vivo assays can be performed. Biochemical assays may be useful to detect miRNA-mediated changes in signaling pathways. Moreover, cell capability of proliferation, differentiation, and migration, as well as receptor binding, can be analyzed in different cellular models [68]. Importantly, miRNA mimic or inhibitor transfection produces not only direct effects on target protein levels, but may also indirectly induce phenotypic changes [65].

A wide range of miRNA-dependent biological effects can also be demonstrated carrying out in vivo experiments. Administration of miRNA mimics carried by adeno-associated viruses (AAV) or lipid-based nanoparticles, in which miRNA mimics are packaged, represent good alternatives to induce an increased miRNA activity in animal models. miRNAs silencing, by contrast, can be obtained by infusion of lipid-based nanoparticles or cholesterol-based compounds, in which antagomirs are packaged.

### 3.2. High-Throughput Technologies

#### 3.2.1. Transcriptomic Analysis and Sequencing

Even though reporter gene assay represents the best strategy to mimic predicted miRNA-mRNA interactions in vivo, one has to keep in mind that results are not completely free of side effects due to transfection [80,81,82], and other disadvantages related to this method can be mentioned:

(1) An artificial increase of miRNA levels over the physiological range may be induced by miRNA mimics, leading consequently to artificial miRNA-target pairing [65,80,81].
(2) miRNAs may alter the activity of several transcription factors, and in this way indirectly affect miRNA-mRNA interaction [42,83].
(3) These assays are time consuming and labour intensive.
(4) Accessibility to mRE sites by miRNA may be very difficult when 3′-UTR sequence gives rise to a complicated secondary structure [84].
(5) The choice of a cell culture as a suitable model to clearly detect consequences of miRNA mimic/inhibitor transfection is challenging: it should express appropriate amounts of endogenous miRNA and target gene.

Recently, in order to overpass the aforementioned limitations, High-Throughput methods have been applied to validate most of the predicted targets.

Next-Generation Sequencing technologies provide new opportunities in the field of miRNA research. In particular, as previously mentioned, RNA-seq is of greater sensitivity and has a larger dynamic range when compared to microarray-based approaches [85]. It offers the possibility of investigating the genome widely in a more precise manner, with the advantages of high throughput, high sensitivity, and high speed. However, the analysis of large-scale data created by this method harbors challenges [86].

Another sequencing-based approach is known as “translational profiling strategy”: in this case, ribosome-associated mRNAs are studied. Only actively translated mRNAs are bound to the ribosome and their sequencing may allow identifying the direct effects of miRNA-induced translational repression [70]. Although this method does not directly measure protein levels, it provides quantitative data in a much more accurate manner than some proteomic approaches, providing a powerful strategy to evaluate miRNA activity [84].

Notwithstanding this, it is not always easy to determine whether the effects of miRNA activity are mainly detected at mRNA or protein level. To solve these issues and to better study miRNA-mRNA complex, some biochemical assays based on co-immunoprecipitation of RISC have been developed.

#### 3.2.2. Biochemical Assays

In this type of approach, after transfecting cells with miRNA mimics or miRNA inhibitors, highly selective antibodies are used to immunoprecipitate the Ago proteins. Then, sequencing is employed to identify miRNAs and mRNAs stably associated with RISC, together with Ago complexes and key proteins that are crucial for RISC function [87,88,89,90].

Disadvantages, such as identifying artificial mRNA-miRISC associations or missing weak interactions between miRNAs and mRNAs, have been recently solved by methods that use UV light to generate a crosslink between RNA and RNA-binding proteins before immunoprecipitation. Among these, HITS-CLIP (High-Throughput sequencing of RNA isolated by crosslinking immunoprecipitation) uses UV irradiations at 254 nm to generate a RNA-miRNA-Ago complex before immunoprecipitation with Ago-specific antibodies. RNA not incorporated in the complex is degraded, while RNA of interest is purified and then subjected to deep sequencing [64]. Thanks to UV light, HITS-CLIP produces much more reproducible miRNA targets rather than immunoprecipitation alone [91]. Importantly, it represents a good technology to validate the interaction of a given miRNA with its mRE target in vivo [65].

PAR-CLIP (photoactivatable-ribonucleoside-enhanced CLIP) is an alternative procedure to HITS-CLIP. It uses 365 nm UV light and 4-thiouridine (or analogs), which is randomly incorporated into RNA during transcription, causing thymidine to cytidine transitions (T to C). Deep sequencing can then reveal where transitions occur. T to C transitions are significantly higher in crosslinked RNA [92], mapping more accurately miRNA-mRNA interactions when compared to HITS-CLIP [70,93].

Additional refinements of the method have led to iCLIP (individual nucleotide resolution CLIP), which uses deletions to drastically increase the resolution of pairing between miRNA sequence and target sites, up to individual nucleotide resolution [94]. Further developments have brought to the crosslinking, ligation, and sequencing of hybrids (CLASH) method, where a ligation step has been added to CLIP procedure. Once miRNA and its target sites within the miRISC complex are ligated, the hybrids are sequenced. However, CLASH efficiency is quite low, so further improvements are necessary to define miRNA-target site with a higher resolution [95].

There are some drawbacks inherent to the immunoprecipitation-based approaches. First of all, they are time-consuming procedures. Then, these strategies identify the interactions between miRNAs and their targets, but do not have a sufficient resolution to perfectly define the mREs. Improved strategies need to be developed in order to experimentally identify miRNA-specific models of interaction with targets and improve miRNA target prediction.

#### 3.2.3. Proteomic Analysis

High-Throughput proteomic methods are available as an alternative to conventional mass spectrometric analysis. In the same way as microarrays search for differences in gene expression, pulsed stable isotope labeling with amino acids in cell culture (pSILAC) allows detecting differences at the protein level [70]. This methodology measures changes in protein production in cells transfected with miRNAs, which have been grown in medium-containing amino acids labelled with light or heavy isotopes. MALDI (matrix-assisted laser desorption/ionization) technology is able to detect the ratio between the light/heavy isotope signals of proteins purified from gels, which seem to be altered due to higher miRNA expression [75,96]. This quantitative method provides a wide view of how miRNAs regulate gene expression at different stages of cell cycle. However, it does not discriminate direct and indirect miRNA targets and does not provide indications about the real site of interaction between miRNA and mRNA [96].

A different but appropriate proteomic approach for validation of miRNA targets is represented by the two-dimensional differentiation in-gel electrophoresis (2D-DIGE), which separate proteins according to isoelectric point and size. If different fluorophores are employed for labeling proteins of different samples, then fluorescent spots obtained from electrophoresis can be analyzed by MALDI. The aim of this strategy is to compare cells with increased or reduced miRNA activity to control cells (for example, cells transfected with a non-targeting control oligonucleotide or treated with scrambled miRNA sequences). This approach has successfully identified a direct target of miR-21 in cancer [79,97].

**Figure 5:**
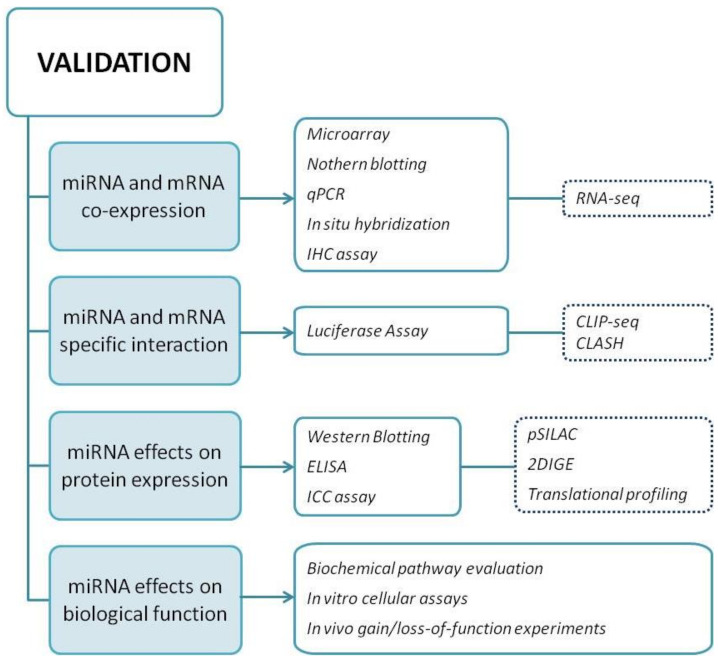
Summary of miRNA validation approach. In the flowchart, all experimental procedures that need to be performed in order to meet the four criteria required for miRNA validation are indicated. Dotted rectangles indicate methods belonging to High-Throughput technologies.

A flowchart summarizing the key approaches for miRNA validation, including all the aforementioned experimental techniques, is shown in Figure 5.

### 3.3. Strengths and Limitations

Compared with the prediction tools, the availability of methods to experimentally verify miRNA targets remains even more challenging [75].

High-Throughput methods may serve as a bridge between computational prediction and experimental validation. They provide higher resolution in detecting true miRNA-mRNA interactions and determining miRNA-induced effects. However, the deep sequencing not only requires time and expensive equipment, but also leads to identify several miRNAs whose biological role is still not known [70,96] and generate a large amount of data that is not easy to interpret. Anyway, High-Throughput data needs to be confirmed by using conventional techniques to determine specific miRNA-mRNA interactions [85].

Predicted interactions are commonly validated by Microarrays, qRT-PCR, Western blots, Luciferase assays, and some HT-methods including CLIP-seq and CLASH [6].

Despite strengths and weaknesses characterizing each strategy, the difference between transcriptomic and proteomic outcomes is crucial. Transcriptomic procedures search for mRNAs of interest and cannot detect repressed targets; on the other hand, proteomic procedures fail to differentiate cleavage from repression events, but certainly do not miss any regulatory effect [6].

Moreover, it is important to remember that gene expression and biochemical approaches investigate two different aspects of miRNA function. The first strategy focuses on analysis of regulatory outcome (at mRNA or protein level), whereas biochemical assays aim to identify miRNA targets, with less consideration toward regulatory outcome [78].

## 4. Conclusions

In recent years, the study of miRNAs has raised growing interest and miRNA regulatory function has been extensively investigated. Research of miRNA target genes has proved to be more complicated than predictable, so it is necessary to continue working to discover the complex rules governing the interaction between miRNAs and their targets. This has supported the development of several computational tools for miRNA target prediction, based on different strategies. Among these, the Machine Learning approaches learn from sample datasets and use the acquired knowledge to make predictions about unknown data. An integration of prediction algorithms based on different rules may significantly improve the prediction accuracy and reduce the number of potential targets. All candidate miRNA targets must be experimentally validated to verify their role. A functional miRNA-mRNA pair must fulfill four criteria. First, both miRNA and the mRNA target gene must be expressed. Second, the miRNA-mRNA interaction must be verified. Third, the effect of miRNA on target protein expression must be demonstrated by using miRNA mimics and miRNA inhibitors. Fourth, miRNA regulation of target gene expression should be related to modified biological functions. Several different methods can be used to perform the validation. High-Throughput methods may be an important resource for this kind or research. Also in this case, computational approaches, which work extremely well in the analysis of genomic, transcriptomic, and proteomic data, could provide precious tools for handling all these data.

The availability of confidently validated miRNA targets is of fundamental importance for the development of effective strategies for miRNA target identification. All the prediction tools are based on, or learn from, verified miRNA-mRNA interactions; thus, they can be improved only by obtaining more validated experimental data. On the other hand, effective and performant predictive tools may provide reliable predictions and allow limiting the number of targets to validate. In the future, it will be more and more necessary to integrate the results obtained from prediction tools and experimental validation, to build a virtuous circle that will aid in improving and deepening our knowledge of miRNA role, action, and network, in physiological and pathological conditions.

## Figures and Tables

**Table 1:** List of miRNA target prediction tools and used strategies. SF: Sequence Features; TS: Thermo-Stability; EC: Evolutionary Conservation; SA: Site Accessibility.

Tool Name	SF	TS	EC	SA	Web	Ref.
miRanda	**√**	**√**	**√**		http://www.microrna.org/microrna	[19]
TargetScan	**√**	**√**	**√**	**√**	http://www.targetscan.org/vert_72	[29]
RNAhybrid		**√**			https://bibiserv.cebitec.uni-bielefeld.de/rnahybrid	[17]
PITA		**√**		**√**	https://genie.weizmann.ac.il/pubs/mir07/mir07_prediction.html	[30]
microTAR	**√**	**√**			http://tiger.dbs.nus.edu.sg/microtar	[16]
PicTar		**√**	**√**		https://pictar.mdc-berlin.de	[27]
PACMIT		**√**	**√**	**√**	https://paccmit.epfl.ch	[31]
MIRZA-G	**√**	**√**	**√**	**√**	http://www.clipz.unibas.ch/index.php?r=tools/sub/mirza_g	[32]
RNA22	**√**	**√**			https://cm.jefferson.edu/rna22/Interactive	[33]

**Table 2:** List of ML prediction tools with the kinds of used strategies. For an explanation of the algorithms, see Box 1.

Tool Name	Algorithm	Positive	Negative	Features	Ref.
MBSTar	Random Forest	MiRbase	Randomly generated	sequence, structure	[40]
NbmiRTar	Naïve Bayes	TarBase	Probability Randomization	sequence	[41]
TargetBoost	Genetic Programming	let-7, lin-4, miR-13a, bantam	Random string	sequence	[42]
DeepTarget	RNN	miRecords miRBase	Mocking in alignment	sequence	[43]
TargetMiner	SVM	miRecords	Randomly generated	seed	[44]
DeepMirTar	Autoencoder	miRecords	Mocking in alignment	sequence, structure, energy and other	[45]
DIANA-microT-CDS	microT-CDS algorithm	miRNA regulatory element in both the 3’-UTR and CDS		sequence, structure, energy and other	[46]
miRanda-mirSVR	SVR (similar to SVM)	set from transfection experiments		sequence, structure, energy and other	[47]
